# Energy intake from unhealthy snack food/beverage among 12‐23‐month‐old children in urban Nepal

**DOI:** 10.1111/mcn.12775

**Published:** 2019-06-21

**Authors:** Alissa M. Pries, Nisha Sharma, Atul Upadhyay, Andrea M. Rehman, Suzanne Filteau, Elaine L. Ferguson

**Affiliations:** ^1^ Helen Keller International New York New York; ^2^ Department of Population Health, Faculty of Epidemiology and Population Health London School of Hygiene and Tropical Medicine London UK

**Keywords:** complementary feeding, double burden, Nepal, snack food, sugar‐sweetened beverage

## Abstract

Unhealthy snack food and beverage (USFB) consumption among young children has been noted in many low‐income and middle‐income countries (LMIC), however, there is a lack of information on the contribution of these foods to children's diets in these contexts. This study describes the nutrient profiles and costs of snacks consumed by young children in Kathmandu Valley, Nepal, and assesses the proportion of total energy intake from nonbreastmilk foods (%TEI‐NBF) contributed by USFB and factors associated with high USFB consumption. A cross‐sectional survey was conducted among 745 randomly sampled primary caregivers of children aged 12–23 months. Of 239 unique snack foods and beverages consumed, 180 (75.3%) were classified as unhealthy based on nutrient profiling, with 158 of these being commercially branded. Median cost/100 kcal of USFB was lower as compared with healthy snacks. Ninety‐one percent of children had consumed a USFB in the previous 24 hr, with these foods contributing a mean %TEI‐NBF of 24.5 ± 0.7 among all children. Biscuits (10.8%), candy/chocolate (3.5%), and savoury snacks (3.4%) provided the largest %TEI‐NBF. Children who were older, female, or from the poorest households had significantly higher odds of high USFB consumption, whereas children whose caregivers were of upper caste/ethnicity or had achieved tertiary education had lower odds of consumption than other children. To reduce USFB consumption, interventions should seek to further understand social/cultural drivers of feeding practices, target disadvantaged populations, and ensure caregivers are fully aware of the nutritional quality of food products they choose for their children.

**Key messages**

- Unhealthy snack foods and beverages (USFB) contribute one‐quarter of energy intake from nonbreastmilk foods among children 12–23 months of age in Kathmandu Valley, with most USFB being commercially produced.
- Children of caregivers from higher wealth status, educational attainment, or upper caste/ethnicity had lower odds of being high consumers of USFB, whereas child age and sex were correlated with high USFB consumption.
- There is a need to target interventions among disadvantaged populations where USFB consumption is highest. There is also a need to ensure that Nepali caregivers are informed about the nutritional quality of commercially produced foods, and that decisions around child‐feeding are free from marketing influence.

## 1 INTRODUCTION

Alongside the growing global availability of processed foods, consumption of unhealthy snack foods and beverages (USFB) has become increasingly prevalent in diets of young children in low‐income and middle‐income countries (LMIC; Faber & Benade, 2007; Pantoja‐Mendoza, Meléndez, Guevara‐Cruz, & Serralde‐Zúñiga, 2015; Pries et al., 2016; Woo et al., 2013). In populations undergoing economic transition, the presence of processed and ultra‐processed foods in diets is increasing (Monteiro, Moubarac, Cannon, Ng, & Popkin, 2013; Popkin, Adair, & Ng, 2012). For young children, the incorporation of ultra‐processed foods into diets introduces foods typically high in sodium/sugar/unhealthy fats at an age when taste preferences are being established (Birch & Doub, 2014; Luque et al., 2018) and could potentially displace consumption of more nutrient‐dense foods (Maunder, Nel, Steyn, Kruger, & Labadarios, 2015). Despite evidence on the increasing prevalence of these shifting consumption patterns, there is limited evidence from LMIC on the nutrient profiles and cost of these foods, which are often assumed to be energy‐dense, nutrient‐poor, and inexpensive (Drewnowski, Darmon, & Briend, 2004).

Despite improvements in child health and survival over the past decade, improvement in diet quality among young children in Nepal has been slow. Between 2006 and 2014, the proportion of Nepali children 6–23 months of age achieving a minimum acceptable diet only rose from 29.5% to 35.4% (Na et al., 2017), with limited dietary diversity driving these suboptimal diets in both rural and urban settings (Ministry of Health Nepal, New ERA, & ICF, 2017). USFB consumption is also highly prevalent among children under 2 years of age in urban Nepal. A 2014 study in Kathmandu Valley found that three‐quarters of children 6–23 months of age had consumed a commercially produced snack food product in the previous day, and snack food product consumption was higher than consumption of many micronutrient‐rich foods, including dark green leafy vegetables, orange‐fleshed fruits and vegetables, and eggs (Pries et al., 2016). Given the low nutrient‐density of many complementary foods in LMIC (Kimmons et al., 2005) and that nearly two‐thirds of children in urban Nepal are not consuming an adequately diverse diet, high consumption of nutrient‐poor snack foods and beverages during the developmentally vital complementary feeding period is concerning. However, there is little information on how much USFB are contributing to dietary intakes among young children, both in Nepal specifically and in LMIC globally (Pries, Filteau, & Ferguson, 2019).

To address these evidence gaps, the aim of this study was to describe characteristics of USFB consumed by children 12–23 months of age in Kathmandu Valley, including their nutrient profiles and costs, and to assess the contribution of USFB to children's energy intakes. Additionally, to build the understanding of factors associated with unhealthy consumption patterns among infants and young children for program design and targeting (Huffman, Piwoz, Vosti, & Dewey, 2014), this study also investigated characteristics of children and their primary caregivers associated with high consumption of USFB.

## 2 METHODS

### 2.1 Study design

A cross‐sectional survey was conducted from February to April 2017 among 745 randomly sampled young children and their caregivers in Kathmandu Valley, Nepal. An electronic interviewer‐administered questionnaire, a paper‐based quantitative multiple‐pass 24‐hr recall (24HR) of children's dietary intake, and anthropometric measurements of mothers were collected. Ethical approval for this study was obtained from the Nepal Health Research Council (reference 563) and the London School of Hygiene and Tropical Medicine (reference 11719).

### 2.2 Study population and sampling

The populations of interest for this study were children 12–23 months of age in Kathmandu Valley and their primary caregivers. The age range of 12–23 months was chosen to cover the nutritionally important complementary feeding period, focusing on older children whose snack consumption was anticipated to be higher than children 6–11 months (Huffman et al., 2014). Primary caregivers were defined as caregivers who provided the majority of care to the child in a day and included: mothers, fathers, grandparents, uncles and aunts, siblings, or a household helper. Children were excluded if they were severely ill on the day of interview, if the child/caregiver did not permanently reside in Kathmandu Valley, or if the child had a congenital/physical malformation that inhibited feeding. Sample size estimations for this survey were calculated based on the primary outcomes of interest for the overall study, which were to assess differences in anthropometrics and micronutrient intakes between high and low consumers of USFB. The calculated sample size for these assessments required a minimum of 702 children.

Children and their caregivers were selected using multi‐stage cluster sampling. For the first stage, 78 clusters were assigned across 1,136 Kathmandu Valley municipality wards based on probability proportionate to size of ward population (CBS, 2012). In total, 68 wards were randomly selected for clusters using a random number generator, with larger wards having more than one cluster assigned. Children and their caregivers in each cluster were recruited 2–3 days prior to scheduled data collection by a trained recruitment team. For each cluster, a random starting GPS point was identified through a sampling grid method (Grais, Rose, & Guthmann, 2007) using government municipality ward maps provided by the Nepal Survey Department. From the GPS point, the first household to the right when facing north was approached to identify an eligible caregiver/child pair. Children and caregivers were first screened for eligibility and then asked if they would like to participate in the study. If more than one eligible child lived in a household or if a child was from a multiple birth, one child would be randomly selected by a random number generator. Caregivers who agreed to participate were provided information on the study procedure and a pictorial dietary tool to aid accurate recall during interview (detailed below). Eleven to twelve caregiver–child pairs per cluster were recruited, with the assumption that several caregivers/children could be unavailable on the day of interview due to illness, family emergency, or change of mind. In the second stage of sampling, all caregivers were contacted on the day of interview and caregivers who were no longer available, or children who were ill, were removed from the sampling frame. Between 9 and 10 child–caregiver pairs per cluster were interviewed; if more than 10 child–caregiver pairs were available in a cluster on the day of data collection, 10 were randomly sampled for interview. Interviews were conducted in the caregivers' homes to ensure a comfortable environment and to aid portion size estimation through use of each household's own utensils for the 24HR. Informed written consent was obtained from all caregivers prior to the interview.

### 2.3 Questionnaire, dietary assessment, and anthropometric measurements

Interviewers first administered the questionnaire and then conducted the 24HR, after which, caregivers who were mothers were brought to a central location for anthropometric measurements. Data were collected on demographic and socio‐economic characteristics pertaining to the caregiver and child, including: caregiver age, educational attainment, parity, asset ownership, caste/ethnic group (including upper castes [e.g., Brahman/Chhetri]; relatively advantaged janajatis [e.g., Newar, Gurung]; disadvantaged non‐dalit Terai caste groups [e.g., Thakur/Yadav]; disadvantaged janajatis [e.g., Magar/Tamang]; and dalits), religion, living conditions, and child age and sex. The questionnaire collected data on additional factors related to child nutrition, including: breastfeeding, food security (Coates, Swindale, & Bilinsky, 2007), and child morbidity in the 24 hr and 2 weeks prior to interview. The questionnaire was translated into Nepali, back‐translated, and pretested prior to data collection. Height and weight of mothers were measured by trained nurses using standardized procedures (Cogill, 2003), and calibrated height measuring boards (Shorr Boards) and SECA digital scales (model 878 U) with ±0.1 kg precision. Two serial measures for height and weight were taken; if the two measures of height differed by more than 0.5 cm or if weight measures differ by more than 0.5 kg these results were discarded and two more serial measures taken.

An interactive 4‐pass 24HR was conducted with each caregiver to obtain information on all foods/beverages consumed by their child over the previous day (Gibson & Ferguson, 2008), including information on the time of day each food/beverage was consumed and who fed the child. Recalls were conducted on all days of the week/weekend to account for day‐of‐the‐week effect at the group level. Portion sizes were estimated using food models and household utensils, with individual recipes and measurements of all ingredients collected for mixed dishes. Portion size estimations were weighed using digital scales (Tanita Model KD‐810) with ±1 g precision. To standardize details collected for foods/beverages and portion size measurements, a probe and portion size guide was developed for interviewers. Caregivers were also asked to recall brands/flavours of commercial products fed to the child, and interviewers verified responses against product packaging available in the household. Additionally, based on formative research, a pictorial dietary recall‐aid was developed for caregivers to reduce recall error. Foods commonly consumed by young children (porridge, eggs, milk, biscuits, savoury snacks, candy, fruits, etc.) were presented pictorially with a grid of times across the day. Caregivers were provided the recall‐aid 2–3 days in advance of the interview, and instructed to tick any foods/beverages provided to the child during the day that would be recalled. On the day the interview, the recall‐aid was collected by interviewers prior to recall, then reviewed by interviewers after the caregiver recalled all foods/beverages to clarify any recall omissions/inclusions. All tools for the 24HR were pilot‐tested among caregivers of children 12–23 months of age during a 2‐week period in December 2016; pilot‐testing was conducted in municipality wards that were not sampled for the survey to avoid bias.

Three market surveys were conducted in September 2016 and May 2017 (for commercial snack food products) and in March 2017 (for non‐commercial snack foods). Collection of costing data for non‐commercial foods was conducted during the survey to ensure real‐time costs (which could vary during other agricultural seasons), whereas the cost of commercial food products was assumed to be steady across time. Costs of commercial snack food and beverage products were collected at 15 points‐of‐sale frequented by caregivers of varying socio‐economic status across all three districts of Kathmandu Valley, including small corner stores, medium independent stores, and large national chain supermarkets. One sample of each product was also purchased for the nutrient content on labels. Nutrient information for commercial products was extracted from labels, and incorporated in the FCT used for analysis (detailed below). Costing data for non‐commercially produced food/beverages (including fruits, milk, grains, legumes, sugar, and eggs) was collected from 11 local markets across Kathmandu Valley covering all three districts. Costs across stores/markets were averaged for each food/beverage in Nepali rupees (NPR), which were then used to calculate the cost per 100 kilocalories (kcal) for each food/beverage as an appropriate food price metric when considering nutritional quality of diets (Jones & Monsivais, 2016). Median costs/100 kcal of foods and inter‐quartile ranges were calculated for categories of foods/beverages.

### 2.4 Data management

Data from the structured interviews were collected electronically on Samsung tablets using the open‐source online platform ONA and Open Data Kit application, with completed questionnaires submitted to the ONA platform each night. Dietary data from the 24HR were collected on paper forms and thoroughly reviewed by a supervisor after each interview. Data from the paper dietary forms were then entered into Microsoft Excel, and the quantities consumed for each food/beverage were calculated using food‐model conversion factors calculated specifically for this study. These conversion factors were developed to convert food model quantities to estimated raw weights of foods/beverages consumed. An average recipe for each mixed dish was also calculated for use in cases where the primary caregivers interviewed had not been present at the time of food preparation/feeding, and individual recipes/portion sizes were not collected for that child.

A food composition table (FCT) was compiled, following guidelines from the Food and Agriculture Organization's International Network of Food Data Systems. This compiled FCT‐used values from: relevant published FCT (Mahidol University, Institute of Nutrition, 2014; Nepal Government, 2012; Public Health England, 2015; Shaheen et al., 2013; US Department of Agriculture, 2015), from nutrient content information on product labels, and from analysed food samples. Fifteen of the most commonly consumed packaged food products were analysed for energy and nutrient (Ca, Fe, Na, Vitamin A, total fat, sugar, carbohydrate, and protein) content. Retention factors were applied to account for micronutrient losses from cooking preparation (Nutrient Data Laboratory, 2002; Shaheen et al., 2013).

As the focus of this study was snack foods and not the act of snacking, the definition of “snacks” was based on categorization of specific food types, not the time of consumption (Johnson & Anderson, 2010; Leech, Worsley, Timperio, & McNaughton, 2015). Snack foods and beverages included foods/beverages commonly referred to in previous literature as snacks, including: biscuits, chocolates/candy, bakery items, savoury chips/crisps, and sugar‐sweetened beverages (soft drinks and juice drinks). Additional Nepal‐specific snack foods were identified through formative research, which included milk, chocolate/malt‐powder‐based drinks, tea, fruits, eggs, breakfast cereals, commercial infant cereal, homemade *jaulo* (porridge made of rice and legumes), and homemade *lito* (infant cereal made of grains/legumes flour). Within this overall category of snack foods or beverages, foods were then subcategorized as “unhealthy” or “healthy,” using a nutrient profiling model from the UK's Food Standards Agency (UK FSA; Food Standards Agency, 2009), which was developed to guide regulation on marketing of unhealthy foods to children. The UK FSA model evaluates the presence and degree of “negative” nutrients (energy, total sugar, saturated fat, and sodium per 100 g; WHO, 2010) and “positive” nutrients (fibre and protein per 100 g, and % fruit/vegetable/nut) to categorize foods as unhealthy or healthy. For powdered products requiring reconstitution, the nutrient profile score was calculated per 100 g of reconstituted product. Two snack food/beverages that were categorized as unhealthy—whole fat milk and egg yolk—were excluded from this category based on global feeding recommendations of animal‐source foods for children below 2 years of age (Pan American Health Organization & WHO, 2001). Terciles of USFB consumption—low/moderate/high consumption—were created based on the proportion of total energy from non‐breastmilk foods (% TEI‐NBF) contributed by snack foods/beverages identified as unhealthy. Total energy intakes from non‐breastmilk foods were calculated based on intake of all foods and beverages reported by caregivers during the 24HR. Whether a child was breastfed or not on the day prior to interview was measured in order to assess breastfeeding status. Quantities of breastmilk intake were not measured, and therefore, total energy intakes reported are based on dietary energy contributions from non‐breastmilk foods only.

### 2.5 Statistical analyses

Data were cleaned and analysed using Stata (version 15). Data and open‐response entries were translated from Nepali to English for analysis. To create quintiles of wealth, relative household wealth status was assessed using a wealth index developed through principal components analysis, including relevant variables related to socio‐economic status: asset ownership, household crowding, home ownership, floor/wall/roof material, source of energy for cooking, and source of drinking water (Vyas & Kumaranayake, 2006). Household food security was assessed based on the Household Food Insecurity Access Scale (Coates et al., 2007). Proportions, means ± standard deviations (SD), and medians with interquartile ranges (IQR) for non‐normally distributed data were calculated to describe the sample and USFB consumption patterns. Two‐sided Pearson's chi‐square tests were used to test differences in proportions, independent sample *t*‐tests or analysis of variance (ANOVA) for differences in means, and Kruskal–Wallis test for differences in medians. Odds ratios and 95% confidence intervals were calculated using ordinal logistic regression for bivariate and multivariable analyses with cluster adjustment. Bivariate analyses explored associations between factors hypothesized to be associated with USFB consumption based on discussions with local experts, prior literature on snack food consumption, and findings from formative research, including: feeding by siblings/secondary caregivers (Sharma et al., 2019), educational attainment (Gatica, Barros, Madruga, Matijasevich, & Santos, 2012; Pries et al., 2016), socio‐economic status (Angeles‐Agdeppa, Lana, & Barba, 2003), maternal anthropometrics (Aitsi‐Selmi, 2015), and child age (Huffman et al., 2014). Final fit of the adjusted model was assessed using manual backward selection and Akaike information criterion. To test the appropriateness of an ordinal regression, the assumption of proportional odds for the dependent variable was assessed using the Brant test. This test was non‐significant for the overall regression model (*p* = 0.215), indicating the assumption was not violated. One independent variable did violate this assumption; removal of this variable from the model did not change the overall model fit or results, and so was excluded from the final model. Collinearity of independent variables was explored through variance inflation factors.

## 3 RESULTS

### 3.1 Description of the study population

**Figure 1:**
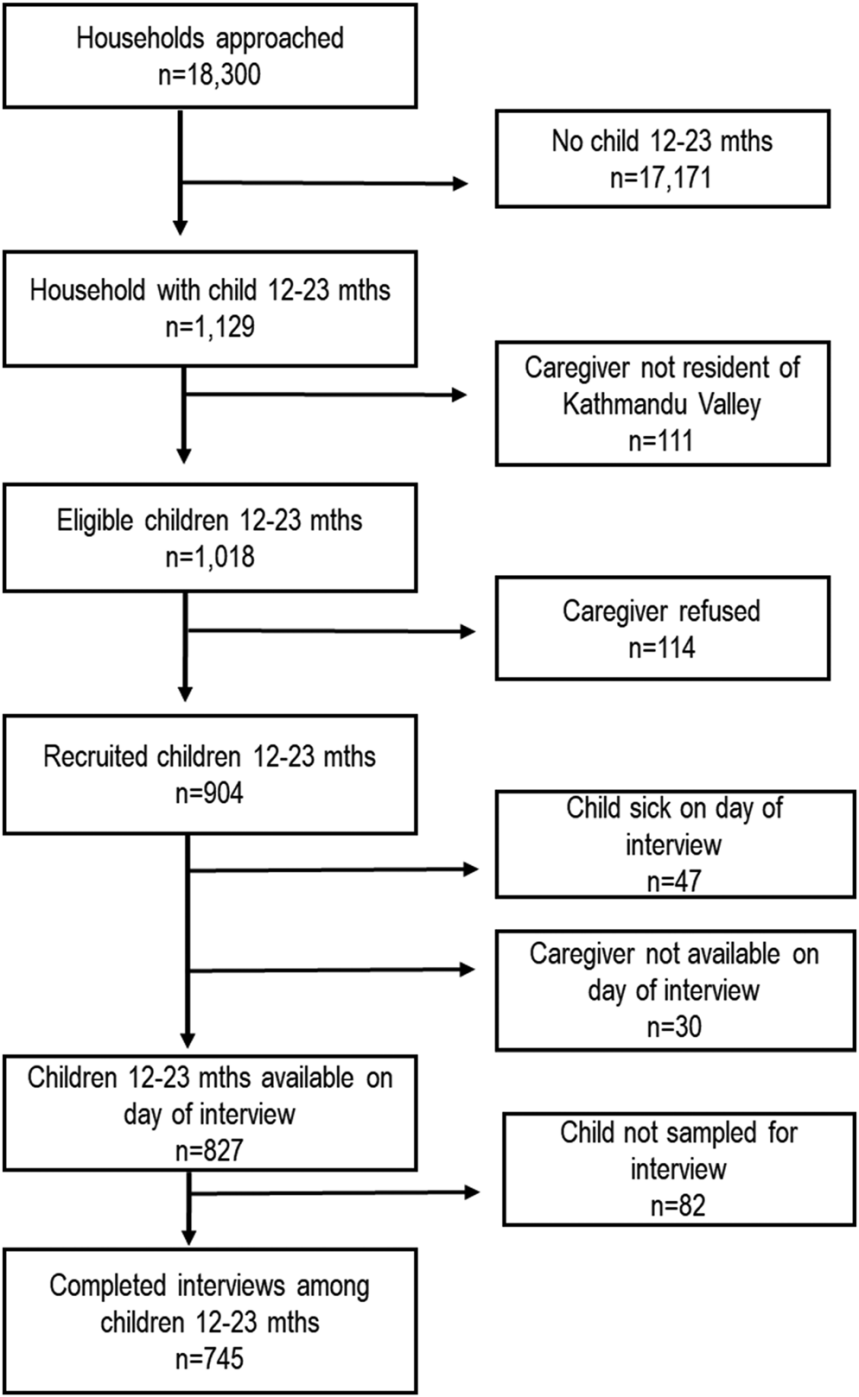
Participant recruitment, exclusion, and inclusion

Results from participant sampling are detailed in Figure 1. Of the 904 child–caregiver pairs recruited, 827 were available for interview on the day of data collection; of the 77 children/caregivers who were not available, 47 were because the child was sick and 30 were because the caregiver was no longer available (most commonly due to visiting relatives or attendance at wedding/festival). The final sample included 745 child–caregiver pairs.

Demographic and socio‐economic characteristics of caregivers, households, and children are presented in Table 1. The majority of caregivers were mothers of the children (90.3%), Hindu (83.4%), and had a mean age of 29 years. Just over half of the children (55.6%) had no siblings, and median household size was 4 members. On average, USFB contributed 24.5% TEI‐NBF across all children, and 5.2%, 21.5%, and 46.9% TEI‐NBF among the lowest, moderate, and highest terciles of USFB consumption, respectively. Comparing socio‐demographics across terciles of USFB consumption showed significant differences related to caregiver religion, caste/ethnicity, education, and wealth status (Table 1). Specifically, as compared with high USFB consumers, a higher proportion of low USFB children had Hindu caregivers (89.2% vs. 76.2%, *p* < 0.001), whereas more high USFB consumers had Buddhist caregivers (17.7% vs. 8.0%, *p* = 0.003). Two‐thirds of caregivers were of either upper caste/ethnicity or advantaged janajati ethnicity. Both low and moderate USFB consumers were more likely to be from an upper caste/ethnicity as compared with high USFB consumers (49.1% vs. 22.6%, *p* < 0.001). More children of caregivers who attended only primary school were high consumers of USFB as compared with low consumers (23.8% vs. 14.1%, *p* = 0.021), whereas more children of caregivers who attended university or postgraduate studies were low USFB consumers as compared with high consumers (24.1% vs. 6.9%, *p* < 0.001). Among households, in the poorest wealth quintile, a higher proportion of children were high USFB consumers as compared with low (28.6% vs. 11.7%, p < 0.001), and a higher proportion of children living in food insecure households were high USFB consumers (18.5% vs. 8.8%, *p* = 0.005).

**Table 1:** Caregiver, household, and child characteristics by unhealthy snack foods and beverages (USFB) consumption tercile^a^^,^ b

	Total (24.5% TEI‐NBF)^c^	Low (5.2% TEI‐NBF)	Moderate (21.5% TEI‐NBF)	High (46.9% TEI‐NBF)
Characteristics	(*N* = 745)	(*N* = 249)	(*N* = 248)	(*N* = 248)
CAREGIVER				
Relationship to child				
Mother	90.3 (673)	86.3 (215)^a^	91.5 (227)^a^	93.2 (231)^b^
Grandmother	7.1 (53)	9.6 (24)	6.9 (17)	4.8 (12)
Other^d^	2.6 (19)	4.0 (10)	1.6 (4)	2.0 (5)
Age (years)				
17–19	2.8 (21)	2.4 (6)	2.0 (5)	4.0 (10)
20–49	92.2 (687)	92.0 (229)	92.3 (229)	92.3 (229)
49–74	5.0 (37)	5.6 (14)	5.7 (14)	3.6 (9)
Religion				
Hindu	83.4 (621)	89.2 (222)^a^	84.7 (210)^a^	76.2 (189)^b^
Buddhist	12.3 (92)	8.0 (20)^a^	11.3 (28)^a^, ^b^	17.7 (44)^b^
Other^e^	4.3 (32)	2.8 (7)	4.0 (10)	6.1 (15)
Ethnic group				
Upper caste	40.3 (300)	58.6 (146)^a^	39.5 (98)^b^	22.6 (56)^c^
Advantaged janajati	26.6 (198)	22.1 (55)	26.2 (65)	31.5 (78)
Disadvantaged janajati	26.2 (195)	15.7 (39)^a^	25.8 (64)^b^	37.1 (92)^c^
Dalit/non‐dalit terai caste	7.0 (52)	3.6 (9)	8.5 (21)	8.9 (22)
Caregiver education				
No formal education	12.8 (95)	13.7 (34)	10.5 (26)	14.1 (35)
Primary	20.3 (151)	14.1 (35)^a^	23.0 (57)^b^	23.8 (59)^b^
Secondary	52.1 (388)	48.2 (120)	52.8 (131)	55.2 (137)
Tertiary	14.9 (111)	24.1 (60)^a^	13.7 (34)^b^	6.9 (17)^b^
Engaged in paid work in the last month	30.9 (230)	23.3 (58)^a^	36.3 (90)^b^	33.1 (82)^a^, ^b^
Engaged in paid work outside the home	16.8 (125)	14.1 (35)	17.7 (44)	18.6 (46)
Maternal nutritional status^f^				
Maternal overweight/obese	42.4 (284)	42.7 (93)	45.1 (101)	39.5 (90)
Maternal underweight	5.5 (37)	6.4 (14)	3.1 (7)	7.0 (16)
HOUSEHOLD^g^				
District of residence				
Kathmandu	68.2 (508)	75.9 (189)^a^	64.9 (161)^b^	63.7 (158)^b^
Lalitpur	22.1 (165)	16.1 (40)^a^	25.8 (64)^b^	24.6 (61)^a^, ^b^
Bhaktapur	9.7 (72)	8.0 (20)	9.3 (23)	11.7 (29)
Male head of household	69.4 (517)	72.7 (181)	68.6 (170)	66.9 (166)
Migration of household member	20.7 (154)	20.1 (50)	23.0 (57)	19.0 (47)
Food secure household	86.4 (644)	91.2 (227)^a^	86.7 (215)^a^, ^b^	81.5 (202)^b^
Household wealth				
Wealthiest	20.0 (149)	23.3 (58)	21.4 (53)	15.3 (38)
Fourth	20.0 (149)	24.5 (61)	16.9 (42)	18.6 (46)
Middle	20.0 (149)	22.1 (55)	22.2 (55)	15.7 (39)
Second	20.0 (149)	18.5 (46)	19.8 (49)	21.8 (54)
Poorest	20.0 (149)	11.7 (29)^a^	19.8 (49)^a^	28.6 (71)^b^
CHILD				
Age (months)				
12–17	56.1 (418)	69.9 (174)^a^	53.2 (132)^b^	45.2 (112)^b^
18–23	43.9 (327)	30.1 (75)^a^	46.8 (116)^b^	54.8 (136)^b^
Sex, female	47.1 (351)	40.6 (101)	50.8 (126)	50.0 (124)
Has a sibling living in household	44.4 (331)	41.4 (103)	50.0 (124)	41.9 (104)
Currently breastfed	91.1 (679)	91.2 (227)	89.5 (222)	92.7 (230)
Morbidity				
Illness in last 24 hrs	22.4 (167)	19.3 (48)	23.4 (58)	24.6 (61)
Illness in last 2 weeks	66.0 (492)	65.9 (164)	64.5 (160)	67.7 (168)

a Values are per cent(n).

b Differing letters (a,b,c) indicate difference between groups at *p* < 0.05 based on ANOVA with Bonferroni post‐hoc test.

c TEI‐NBF: total energy intake from non‐breastmilk foods.

d Other caregiver types included: aunt, father, house helper, cousin, and grandfather.

e Other religions included: Christian, Kirat, and Muslim.

f Of caregivers who are mothers; *n* = 670.

g Household of child.

### 3.2 Description of USFB consumption

A total of 239 unique snack foods or beverages were consumed among the children 12–23 months of age, with 180 (75.3%) of these foods or beverages categorized as unhealthy based on nutrient profiling. Biscuits made up a large proportion of snack foods consumed by the children, with 73 unique biscuit products consumed across all children. Of the 180 USFB, 87.8% (*n* = 158) were commercially produced/branded, with the remaining sold by a vendor/shop but not branded. All snacks consumed by the children, healthy and unhealthy, provided on average 54.2%TEI‐NBF, with USFB providing nearly half of this contribution to dietary energy intake (24.5%TEI‐NBF). The %TEI‐NBF by categories of USFB and their prevalence of consumption among all children are detailed in Table 2. Though unhealthy snack beverages were consumed by nearly one‐third of all children, unhealthy snack foods provided a far greater contribution to TEI‐NBF (22.5% vs. 2.0%, respectively). The most commonly consumed categories of unhealthy snack foods were biscuits, candy, and savoury snacks, which also contributed the greatest %TEI‐NBF (3–11%). Fruit juice drinks were consumed by less than 10% of all children, but provided the largest %TEI‐NBF of all unhealthy snack beverages. Median intakes of saturated fat, sugar, and sodium among all children were 8.7 g (IQR: 4.8–14.2 g), 28.4 g (IQR: 17.2–44.1 g), and 250 mg (IQR: 152‐407 mg), respectively, with USFB providing an average of 30.9% of total saturated fat, 31.1% of total sugar, and 44.9% of total sodium intakes among all children.

**Table 2:** Consumption of unhealthy snack foods and beverages (USFB) and contribution to intakes of energy, sugar, sodium, and saturated fats^a^

Food categories	Consumption by children	% TEI‐NBF^b^	% Total sugar	% Total sodium	% Total saturated fat
*ALL USFB*	91.0 (678)	24.5 ± 0.7	31.1 ± 1.0	44.9 ± 1.1	30.9 ± 1.0
*UNHEALTHY SNACK FOODS*	89.7 (668)	22.5 ± 0.7	22.8 ± 0.8	44.3 ± 1.1	30.9 ± 1.0
Biscuits	68.6 (511)	10.8 ± 0.5	10.7 ± 0.5	20.7 ± 0.9	13.1 ± 0.6
Candy/chocolates	55.2 (411)	3.5 ± 0.2	8.5 ± 0.5	1.8 ± 0.2	6.1 ± 0.4
Savoury snacks	39.7 (296)	3.4 ± 0.3	0.7 ± 0.1	10.3 ± 0.7	5.4 ± 0.4
Instant noodles	16.8 (125)	2.2 ± 0.2	0.3 ± 0.05	7.0 ± 0.7	3.1 ± 0.3
Sweet bread/bakery	12.6 (94)	2.0 ± 0.2	1.8 ± 0.3	3.1 ± 0.4	2.5 ± 0.3
Traditional savoury snacks	4.2 (31)	0.2 ± 0.04	0.1 ± 0.03	0.7 ± 0.2	0.2 ± 0.06
Processed dairy^c^	1.7 (13)	0.2 ± 0.09	0.2 ± 0.1	0.2 ± 0.07	0.2 ± 0.09
Sugary breakfast cereal	1.7 (13)	0.1 ± 0.03	0.1 ± 0.03	0.3 ± 0.1	0.1 ± 0.01
Traditional sweet snacks	1.6 (12)	0.1 ± 0.04	0.4 ± 0.2	0.2 ± 0.09	0.2 ± 0.1
*UNHEALTHY SNACK BEVERAGES*	31.3 (233)	2.0 ± 0.2	8.3 ± 0.6	0.6 ± 0.09	0.0 ± 0.0
Sweetened tea/water	22.0 (164)	0.8 ± 0.08	4.7 ± 0.4	0.1 ± 0.01	0.0 ± 0.0
Fruit juice drinks	8.9 (66)	1.0 ± 0.1	2.9 ± 0.4	0.3 ± 0.06	0.0 ± 0.0
Soft drinks	2.7 (20)	0.1 ± 0.04	0.5 ± 0.1	0.1 ± 0.03	0.0 ± 0.0
Chocolate‐powder drinks	1.2 (9)	0.1 ± 0.04	0.2 ± 0.09	0.1 ± 0.05	0.0 ± 0.0

a Values presented as n(%) and mean ± robust standard error.

b TEI‐NBF: total energy intake from non‐breastmilk foods.

c Included ice cream and sweetened curd.

Consumption of USFB most commonly occurred in the morning (before 10 am) and afternoon (between 2 and 6 pm), with 64.8% (*n* = 483) and 66.2% (*n* = 493) of children consuming USFB at these times, respectively. Forty‐one percent of children (*n* = 306) consumed USFB mid‐day (10‐2 pm) and one‐quarter (26.4%, *n* = 197) consumed them in the evening (after 6 pm). Children were typically fed USFB by their primary caregiver, but 32.6% (*n* = 243) of children were also fed USFB by a secondary caregiver. The most common secondary caregivers feeding USFB were fathers (9.7%, *n* = 72), grandmothers (8.5%, *n* = 63), siblings (7.8%, *n* = 58), and aunts (7.8%, n = 58). There was no significant difference in the proportion of high USFB consumers who were fed any food from secondary caregivers as compared with low USFB consumers (44.8% vs. 37.8%, *p* = 0.286).

**Figure 2:**
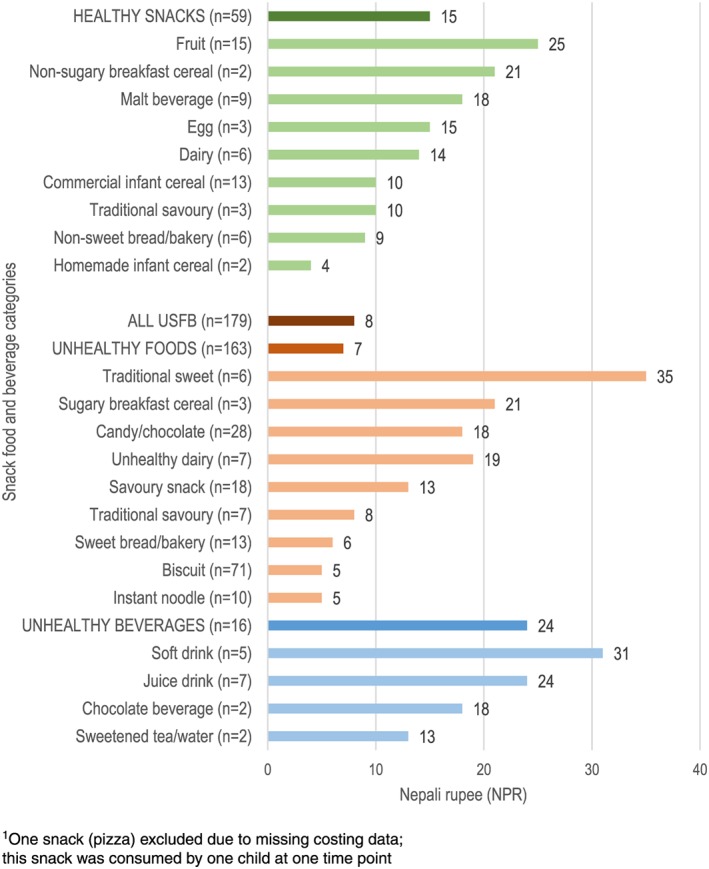
Median cost/100 kcal of unhealthy snack foods and beverages (USFB) and healthy snack foods^1^

Median cost/100 kcal of healthy and unhealthy snack foods and beverages are presented in Figure 2. The median cost for USFB was 8 NPR/100 kcal versus 15 NPR/100 kcal for healthy snack foods and beverages (*p* < 0.001). Biscuits, which provided the largest %TEI‐NBF among all USFB, were the least expensive USFB. The median expenditure per child on total kcal of USFB consumed was 10 NPR, with no difference noted across wealth quintiles (*p* = 0.247). There was also no difference in cost/100 kcal of USFB consumed across wealth groups (*p* = 0.060). The median expenditure per child on total kcal of healthy snack foods/beverages consumed was 22 NPR, with the poorest households spending a median of 13 NPR and the wealthiest households spending 30 NPR (*p* < 0.001). Median cost/100 kcal of USFB consumed was also significantly higher among the wealthiest households, as compared with the poorest households (14 vs. 12 NPR/100 kcal, *p* < 0.001).

### 3.3 Factors associated with high consumption of USFB

In the adjusted model, children who were female, 18 months or older, or from the poorest households were more likely to be high consumers of USFB, whereas children of caregivers who had higher educational attainment or were of an upper caste/ethnicity were less likely to be high USFB consumers (Table 3). Comparison of %TEI‐NBF from USFB categories by child sex and age, and caregiver caste/ethnicity, wealth status, and educational attainment, are presented in Tables S1–S5. Female children had significantly higher %TEI‐NBF from unhealthy snack foods, but not unhealthy snack beverages, as compared with male children. Child age, wealth status, educational attainment, and caste/ethnicity showed an association with %TEI‐NBF from both unhealthy snack foods and unhealthy snack beverages. Children in the poorest households had higher %TEI‐NBF from biscuits and savoury snacks, as compared with children in higher wealth quintiles (*p* = 0.002 and *p* = 0.005, respectively). Children of caregivers who achieved tertiary education had lower %TEI‐NBF across all categories of USFB.

**Table 3:** Ordinal logistic regression model for high consumption^a^ of unhealthy snack foods and beverages (comparison with low/moderate consumption^b^)

	Unadjusted			Adjusted		
	OR^c^	95% CI^d^	P	OR	95% CI	p
*District of residence*						
Kathmandu	1	‐	0.027	1	‐	0.061
Lalitpur	1.52	1.08–2.13		1.49	0.95–2.33	
Bhaktapur	1.52	0.97–2.41		1.43	0.97–2.10	
Caregiver relationship to child, mother	1.83	1.15–2.90	0.010			
*Caregiver age*						
17–19 (ref)	1	‐	0.255			
20–49	0.63	0.29–1.39				
49–74	0.47	0.19–1.15				
Religion, Hindu	0.49	0.34–0.70	<0.001			
*Ethnic group*						
Upper caste (ref)	1	‐	<0.001	1	‐	<0.001
Advantaged janajati	2.66	1.96–3.59		2.55	1.80–3.63	
Disadvantaged janajati	3.82	2.73–5.34		2.98	2.14–4.16	
Dalit/non‐dalit terai caste	3.52	2.18–5.68		2.61	1.53–4.44	
*Caregiver education*						
Tertiary (ref)	1	‐	<0.001	1	‐	0.040
Secondary	2.73	1.77–4.22		1.93	1.21–3.06	
Primary	3.51	2.23–5.53		1.77	1.06–2.96	
No formal education	2.53	1.43–4.49		1.45	0.79–2.67	
Worked in the last month	1.40	1.03–1.89	0.030			
Works outside the home	1.27	0.88–1.84	0.208			
Male head of household	0.82	0.62–1.08	0.152			
Migration of household member	0.95	0.70–1.29	0.745			
Food secure household^b^	0.53	0.38–0.76	<0.001			
*Household wealth index*						
Wealthiest (ref)	1	‐	<0.001	1	‐	0.052
Fourth	1.06	0.69–1.63		1.11	0.75–1.64	
Middle	1.06	0.68–1.68		1.16	0.72–1.84	
Second	1.53	0.96–2.43		1.53	0.92–2.54	
Poorest	2.58	1.63–4.06		2.15	1.29–3.60	
*Child age*						
12–17 months (ref)	1	‐	<0.001	1	‐	<0.001
18–23 months	2.14	1.65–2.79		2.06	1.57–2.71	
Child sex, female	1.33	1.04–1.70	0.024	1.35	1.06–1.73	0.016
Sibling living in household	1.02	0.80–1.30	0.885			
Child illness in last 24 hours	1.26	0.94–1.67	0.120			
Currently breastfed	1.15	0.72–1.83	0.553			

a High consumption: children in highest tercile consumption from USFB (mean 46.9% total energy intake from non‐breastmilk foods [TEI‐NBF]).

b Low consumption: children in lowest tercile of consumption from USFB (mean 5.2% TEI‐NBF); moderate consumption: children in middle tercile of consumption from USFB (mean 21.5%‐NBF).

c OR: odds ratio.

d CI: confidence interval.

## 4 DISCUSSION

This study among 12–23 month old children in Kathmandu Valley indicates that the majority of snack foods and beverages consumed by young children are unhealthy according to their nutrient profile. Most children had consumed USFB in the previous 24 hr, and USFB contributed on average almost half of TEI‐NBF among the highest consumers and one‐quarter of TEI‐NBF among all children. Being female, over 18 months of age, or being from the poorest wealth quintile increased a child's likelihood of high USFB consumption, whereas children from upper caste/ethnicity households or with a caregiver who attained tertiary‐level education had lower odds of being high consumers.

The high %TEI‐NBF contributed by USFB in the diets of 12–23 month olds among urban children in Nepal is alarming. It is comparable with the %TEI from USFB among 12–23 month olds reported in other low‐income and middle‐income settings, which range from 9% to 40%, with a median of 19% (Anderson, Cornwall, Jack, & Gibson, 2008; Denney, Afeiche, Eldridge, & Villalpando‐Carrión, 2017; Jeharsae, Sangthong, & Chongsuvivatwong, 2011; Karnopp et al., 2017; Kavle et al., 2015; Lander et al., 2010; Roche, Creed‐Kanashiro, Tuesta, & Kuhlein, 2011; Rodríguez‐Ramírez, Muñoz‐Espinosa, Rivera, González‐Castell, & González de Cosío, 2016; Valmórbida & Vitolo, 2014), and is also comparable with toddlers and school‐age children in high‐income settings (Kant, 2003; Webb et al., 2006). The higher %TEI‐NBF from USFB among 18–23 month olds as compared with 12–17 month olds observed in this study is consistent with intakes reported in high‐income settings (Hamner, Perrine, Gupta, Herrick, & Cogswell, 2017) and general trends of increased USFB consumption with age in LMIC settings (Denney et al., 2017; Lander et al., 2010), including Nepal (Pries et al., 2016). This relationship likely relates to introduction of new foods/flavours and incorporation of family foods into the diet as a child ages. In addition to increasing the risk for overnutrition in childhood by providing excessive energy intakes (Nicklas, Yang, Baranowski, Zakeri, & Berenson, 2003; Welsh, 2005), early consumption of USFB can establish taste preferences for less healthy foods that continue into later childhood (Luque et al., 2018). Another consequence of high USFB consumption is the potential for displacement of other nutrient‐rich foods (Maunder et al., 2015), which could be detrimental for growth and development among this young age group.

Our study results showing an association between higher wealth status/educational attainment with lower USFB consumption are consistent with other studies in LMIC (Anderson et al., 2008; Bentley et al., 2015; Gatica et al., 2012; Jeharsae et al., 2011; Pries et al., 2016). It has been hypothesized that families in low‐income settings may be more inclined to purchase energy‐dense snack food products because these products could be a more affordable food option (Angeles‐Agdeppa et al., 2003; Drewnowski et al., 2004). Although there was no difference in total amount spent on USFB across levels of wealth in this study, wealthier households spent significantly more on healthy snacks for their children. This may indicate that although the low costs of USFB facilitated use across all wealth groups, or that all children prefer low‐cost USFB, the higher costs of healthier foods may have differentially influenced snack choices by caregivers of varying wealth status. The role of education and provision of snack foods to young children has also been previously explored, with studies noting an inverse relationship between caregiver educational attainment and unhealthy food consumption among children in both high and low/middle income contexts (Gatica et al., 2012; Kranz & Siega‐Riz, 2002). In Nepal, increasing levels of maternal educational attainment have been correlated with improved infant and young child feeding practices (Khanal, Sauer, & Zhao, 2013) and prevalence of commercial snack food consumption in Kathmandu Valley has been found to be lower among young children with mothers who have attended university (Pries et al., 2016). This relationship between higher caregiver education and lower USFB consumption could be related to higher nutritional literacy or understanding of product labels among caregivers with higher levels of education. High USFB consumption among children in low socio‐economic households, where families likely already have limited access to healthy nutrient‐rich foods, highlights an area where more programmatic work is needed to improve complementary feeding in urban Nepal.

The greater odds of high USFB consumption among female children and lower odds among children from upper caste households has not been reported elsewhere; these results indicate that sociocultural beliefs may be influencing diets of young children in Nepal. Although inequitable intra‐household food allocation by sex has been noted in South Asian contexts including Nepal, (Harris‐Fry, Shrestha, Costello, & Saville, 2017), literature on the influence of child sex and feeding of USFB is extremely limited. Fledderjohann et al. (2014) found greater consumption of fresh milk by sons as compared with daughters among Indian children below 5 years of age; further exploration of our data showed that male children consumed a higher %TEI‐NBF from healthy snacks than female children, particularly dairy‐based snacks. No studies exploring sex differentials in feeding of USFB to infants and young children have been identified. Although preference for the birth of a son has been noted in some regions (Puri, 2015), sex‐difference in complementary feeding practices have not been noted in the last decade in Nepal (Na et al., 2017), and it cannot be assumed that differentials in feeding of USFB is a result of gender bias. Female infants in Nepal are typically introduced to solid foods around 1 month earlier than male children during *pasni* (rice feeding ceremony for infants; Tamang et al., 2002), which may tie to a belief that female children can be introduced to a wider range of foods, potentially including USFB, earlier than boys. Sugar contributed a greater %TEI‐NBF among female as compared with male children, illustrated by their greater %TEI‐NBF from candy, indicating that there may be a preference to feed females sweeter foods than males. Innate preference for sweet foods in early childhood is typically similar for both sexes (Ventura & Worobey, 2013), supporting the hypothesis that the difference in USFB consumption by child sex is caregiver‐driven, rather than responding to preferences among female children. Caste/ethnicity also play a strong role in food beliefs and eating practices, which may account for the differences in USFB consumption by ethnic groups in this study and which have been noted in a previous Kathmandu Valley study (Pries et al., 2016). Among upper caste groups, particularly Brahmin, the concept of “purity” influences both eating practices and food restrictions (Subedi, 2010), and such beliefs and practices could be contributing to lower %TEI‐NBF from USFB among children in these households. Achievement of minimum dietary diversity has been found to be lower among young children from disadvantaged societal/ethnic groups (Na et al., 2017), and higher consumption of nutrient‐poor USFB among these children with already limited diet quality is concerning. Further research on caregivers' perceptions of foods among boys vs. girls, and feeding practices across ethnic groups, would provide needed insights for interventions hoping to reduce consumption of USFB during the complementary feeding period.

Nine out of ten USFB items consumed by children in this study were commercially branded food or beverage products. Such products are typically high in sugar/sodium/unhealthy fats, and the USFB consumed by children in Kathmandu Valley provided nearly one‐third and one‐half their total sugar and sodium intakes, respectively, and were primarily commercial biscuits, candy, savoury snacks, instant noodles, and bakery products. Although many processed or ultra‐processed foods are not marketed as intended for infants and young children, they are commonly consumed by children during the complementary feeding period in LMIC (Huffman et al., 2014). As young children are biologically inclined to favour highly sweet or salty foods (Mennella, 2014), and because such products require minimal preparation and are easily self‐fed, caregivers may opt for such foods for their young children because they are perceived to be appealing to the child and convenient to feed (Brunner, van der Horst, & Siegrist, 2010). There is a need for front‐of‐pack labelling to ensure Nepali caregivers are well‐informed about the nutritional quality of commercially produced foods they provide to their young children, and a need for regulations to ensure that their decisions around child‐feeding are free from marketing influence.

This study has several limitations. First, the cross‐sectional design restricts the ability to establish causality of factors associated with high USFB consumption among children. Second, although USFB consumption is typically higher in urban as compared with rural areas (Huffman et al., 2014), by focusing this study in Kathmandu Valley, the extent of USFB in diets of young children in rural areas remains unknown. However, this study's finding that USFB are a major part of diets among young children in urban Nepal is an important one—though the majority of Nepal's population is rural, the country has one of the fastest rates of urbanization globally (United Nations, Department of Economic and Social Affairs (UN DESA), Population Division, 2018) and so health and nutrition of urban populations is increasingly important to consider. Third, although children's current breastfeeding status was assessed, no quantification of breastmilk intake was measured and therefore the energy intakes presented are unable to include specific energy contributed by breastmilk. However, no relationship between breastfeeding status and USFB consumption was found, indicating that intakes were similar for both breastfed and non‐breastfed children. Fourth, because measuring salt intake in dietary assessments is difficult and often results in over‐estimation, our estimates of sodium intake were based on sodium content in foods as per our compiled FCT. However, we analysed the actual sodium content of the 15 most commonly consumed food products to ensure accuracy during analysis. Although salt is not commonly added to infant foods (sugar is more commonly added, and was measured), we note that there could be an underestimation of salt intake in our analysis. However, given typical infant feeding practices in Nepal and our careful consideration of sodium values in foods, it is unlikely that this limitation would change findings. Finally, the nutrient profiling model (UK FSA) used in this study was developed to restrict marketing of unhealthy foods to children of all ages. This model was selected in lieu of any existing models for young children specifically because it has been validated (Arambepola, Scarborough, & Rayner, 2008). Given that children below 2 years of age have different nutrient requirements than older children, there is a need for development of a nutrient profiling model for foods consumed during the complementary feeding period.

Predominantly processed foods that are high in sugar or sodium should be discouraged from regular use in young child feeding and appropriate regulatory measures should be taken so that caregivers are aware of the nutritional quality of commercially produced foods and beverages fed to their children. Given the relationships between caregiver wealth status/educational attainment and high USFB consumption, there is indication that this unhealthy consumption pattern is greatest among populations of lower socio‐economic status; disadvantaged populations should therefore be targeted to improve awareness of the nutritional quality of snacks fed to children. Finally, as programs look towards tackling the growing double burden in LMIC, consideration of how feeding practices and sociocultural beliefs interact with use of USFB for children should be explored.

## CONFLICTS OF INTEREST

The authors declare that they have no conflicts of interest.

## CONTRIBUTIONS

AP, EF, SF, and AR designed the study. AP and NS managed data collection. AR advised on data analysis and AP conducted data analysis. AP developed the manuscript and all authors provided comprehensive review of the manuscript.

## Supporting information

Supplemental table 1. %TEI‐NBF from USFB by child sex^1,2^
Supplemental table 2. %TEI‐NBF from USFB by child age^1,2^
Supplemental table 3. %TEI‐NBF from USFB by caste/ethnicity^1,2^
Supplemental table 4. %TEI‐NBF from USFB by poorest households (wealth quintile 1)^1,2^
Supplemental table 5. %TEI‐NBF from USFB by educational attainment^1,2^
