# Supplementary material for: Energy intake from unhealthy snack food/beverage among 12‐23‐month‐old children in urban Nepal
Source: Matern Child Nutr. 2019 Jun 21;15(Suppl 4):e12775. doi: 10.1111/mcn.12775 (PMC6617731; doi:10.1111/mcn.12775)
Supplement: Supplementary file 1 — Supplemental table 1. %TEI‐NBF from USFB by child sex1,2 Supplemental table 2. %TEI‐NBF from USFB by child age1,2 Supplemental table 3. %TEI‐NBF from USFB by caste/ethnicity1,2 Supplemental table 4. %TEI‐NBF from USFB by poorest households (wealth quintile 1)1,2 Supplemental table 5. %TEI‐NBF from USFB by educational attainment1,2 [file MCN-15-e12775-s001.docx]

| Supplemental table 1. %TEI-NBF from USFB by child sex^1,2^ | | | |  |
| --- | --- | --- | --- | --- |
| Food categories | % TEI-NBF (all)  N=745 | % TEI-NBF (female)  N=351 | % TEI-NBF (male)  N=394 | *p* |
| *ALL USFB* | 24.5 ± 0.7 | 26.3 ± 1.4 | 22.9 ± 1.0 | 0.016 |
| *UNHEALTHY SNACK FOODS* | 22.5 ± 0.7 | 24.3 ± 1.4 | 20.9 ± 0.9 | 0.015 |
| Biscuits | 10.8 ± 0.5 | 10.7 ± 0.9 | 10.9 ± 0.6 | 0.809 |
| Candy/chocolates | 3.5 ± 0.2 | 4.2 ± 0.4 | 3.0 ± 0.2 | 0.006 |
| Savoury snacks | 3.4 ± 0.3 | 4.0 ± 0.5 | 2.8 ± 0.3 | 0.022 |
| Instant noodles | 2.2 ± 0.2 | 2.2 ± 0.5 | 2.1 ± 0.3 | 0.854 |
| Bakery items | 2.0 ± 0.2 | 2.3 ± 0.5 | 1.7 ± 0.3 | 0.285 |
| *UNHEALTHY SNACK BEVERAGES* | 2.0 ± 0.2 | 2.0 ± 0.3 | 1.9 ± 0.2 | 0.823 |
| ^1^Values presented as mean ± robust standard error  ^2^TEI-NFB: total energy intake from non-breastmilk foods | | | | |

| Supplemental table 2. %TEI-NBF from USFB by child age^1,2^ | | | |  |
| --- | --- | --- | --- | --- |
| Food categories | % TEI-NBF (all)  N=745 | % TEI-NBF  (12-17 mth)  N=300 | % TEI-NBF  (18-23 mth)  N=445 | *p* |
| *ALL USFB* | 24.5 ± 0.7 | 21.3 ± 0.9 | 28.6 ± 1.4 | <0.001 |
| *UNHEALTHY SNACK FOODS* | 22.5 ± 0.7 | 19.8 ± 0.9 | 26.0 ± 1.4 | <0.001 |
| Biscuits | 10.8 ± 0.5 | 9.9 ± 0.6 | 12.0 ± 0.9 | 0.027 |
| Candy/chocolates | 3.5 ± 0.2 | 3.2 ± 0.3 | 4.0 ± 0.4 | 0.050 |
| Savoury snacks | 3.4 ± 0.3 | 3.1 ± 0.4 | 3.7 ± 0.5 | 0.224 |
| Instant noodles | 2.2 ± 0.2 | 1.9 ± 0.3 | 2.5 ± 0.5 | 0.165 |
| Bakery items | 2.0 ± 0.2 | 1.2 ± 0.2 | 3.1 ±0.5 | <0.001 |
| *UNHEALTHY SNACK BEVERAGES* | 2.0 ± 0.2 | 1.5 ± 0.2 | 2.6 ± 0.4 | 0.002 |
| ^1^Values presented as mean ± robust standard error  ^2^TEI-NFB: total energy intake from non-breastmilk foods | | | | |

| Supplemental table 3. %TEI-NBF from USFB by caste/ethnicity^1,2^ | | | |  |
| --- | --- | --- | --- | --- |
| Food categories | % TEI-NBF (all)  N=745 | % TEI-NBF (upper caste)  N=300 | % TEI-NBF (non-upper caste)  N=445 | *p* |
| *ALL USFB* | 24.5 ± 0.7 | 17.9 ± 1.3 | 29.0 ± 1.0 | <0.001 |
| *UNHEALTHY SNACK FOODS* | 22.5 ± 0.7 | 16.4 ± 1.3 | 26.6 ± 0.9 | <0.001 |
| Biscuits | 10.8 ± 0.5 | 7.8 ± 0.9 | 12.8 ± 0.6 | <0.001 |
| Candy/chocolates | 3.5 ± 0.2 | 3.0 ± 0.4 | 3.9 ± 0.3 | 0.028 |
| Savoury snacks | 3.4 ± 0.3 | 1.9 ± 0.5 | 4.4 ± 0.4 | <0.001 |
| Instant noodles | 2.2 ± 0.2 | 1.2 ± 0.4 | 2.8 ± 0.4 | <0.001 |
| Bakery items | 2.0 ± 0.2 | 1.8 ± 0.5 | 2.1 ± 0.3 | 0.573 |
| *UNHEALTHY SNACK BEVERAGES* | 2.0 ± 0.2 | 1.5 ± 0.3 | 2.3 ± 0.2 | 0.026 |
| ^1^Values presented as mean ± robust standard error  ^2^TEI-NFB: total energy intake from non-breastmilk foods | | | | |

| Supplemental table 4. %TEI-NBF from USFB by poorest households (wealth quintile 1)^1,2^ | | | |  |
| --- | --- | --- | --- | --- |
| Food categories | % TEI-NBF (all)  N=745 | % TEI-NBF  (wealth quintile 1)  N=149 | % TEI-NBF  (wealth quintiles 2-5)  N=596 | *p* |
| *ALL USFB* | 24.5 ± 0.7 | 32.1 ± 1.9 | 22.6 ± 0.8 | <0.001 |
| *UNHEALTHY SNACK FOODS* | 22.5 ± 0.7 | 29.4 ± 1.8 | 20.8 ± 0.7 | <0.001 |
| Biscuits | 10.8 ± 0.5 | 14.0 ± 1.3 | 10.0 ± 0.5 | 0.002 |
| Candy/chocolates | 3.5 ± 0.2 | 3.9 ± 0.5 | 3.4 ± 0.2 | 0.342 |
| Savoury snacks | 3.4 ± 0.3 | 5.2 ± 0.8 | 2.9 ± 0.3 | 0.005 |
| Instant noodles | 2.2 ± 0.2 | 2.9 ± 0.7 | 2.0 ± 0.2 | 0.172 |
| Bakery items | 2.0 ± 0.2 | 2.9 ± 0.7 | 1.8 ± 0.3 | 0.139 |
| *UNHEALTHY SNACK BEVERAGES* | 2.0 ± 0.2 | 2.7 ± 0.5 | 1.8 ± 0.2 | 0.059 |
| ^1^Values presented as mean ± robust standard error  ^2^TEI-NFB: total energy intake from non-breastmilk foods | | | | |

| Supplemental table 5. %TEI-NBF from USFB by educational attainment^1,2^ | | | |  |
| --- | --- | --- | --- | --- |
| Food categories | % TEI-NBF (all)  N=745 | % TEI-NBF  (tertiary)  N=111 | % TEI-NBF (secondary or lower)  N=634 | *p* |
| *ALL USFB* | 24.5 ± 0.7 | 15.3 ± 1.6 | 26.1 ± 0.8 | <0.001 |
| *UNHEALTHY SNACK FOODS* | 22.5 ± 0.7 | 14.1 ± 1.5 | 24.0 ± 0.7 | <0.001 |
| Biscuits | 10.8 ± 0.5 | 8.7 ± 1.2 | 11.1 ± 0.5 | 0.036 |
| Candy/chocolates | 3.5 ± 0.2 | 2.1 ± 0.5 | 3.8 ± 0.2 | 0.001 |
| Savoury snacks | 3.4 ± 0.3 | 1.6 ± 0.5 | 3.7 ± 0.3 | <0.001 |
| Instant noodles | 2.2 ± 0.2 | 0.9 ± 0.4 | 2.4 ± 0.3 | <0.001 |
| Bakery items | 2.0 ± 0.2 | 0.3 ± 0.3 | 2.3 ± 0.3 | <0.001 |
| *UNHEALTHY SNACK BEVERAGES* | 2.0 ± 0.2 | 1.1 ± 0.4 | 2.1 ± 0.2 | 0.015 |
| ^1^Values presented as mean ± robust standard error  ^2^TEI-NFB: total energy intake from non-breastmilk foods | | | | |
